# Low dose of morphine to relieve dyspnea in acute respiratory failure (OpiDys): protocol for a double-blind randomized controlled study

**DOI:** 10.1186/s13063-022-06754-3

**Published:** 2022-09-30

**Authors:** Alexandre Demoule, Robin Deleris, Côme Bureau, Said Lebbah, Maxens Decavèle, Martin Dres, Thomas Similowski, Agnes Dechartres

**Affiliations:** 1 Service de Médecine Intensive et Réanimation (Département R3S), AP-HP, Groupe Hospitalier Universitaire APHP-Sorbonne Université, site Pitié-Salpêtrière, F-75013 Paris, France; 2 Sorbonne Université, INSERM, UMRS1158 Neurophysiologie Respiratoire Expérimentale et Clinique, F-75005 Paris, France; 3 Département de Santé Publique, Unité de Recherche Clinique Pitié-Sapêtrière-Charles Foix, APHP.Sorbonne Université, site Pitié-Salpêtrière, Paris, France; 4 AP-HP, Groupe Hospitalier Universitaire APHP-Sorbonne Université, site Pitié-Salpêtrière, Département R3S, F-75013 Paris, France; 5 Département de Santé Publique, Sorbonne Université, INSERM, Institut Pierre Louis d’Epidémiologie et de Santé Publique, APHP.Sorbonne Université, Hôpital Pitié Salpêtrière, F75013 Paris, France

**Keywords:** Dyspnea, Opioids, Intensive care, Mechanical ventilation, Randomized controlled trial

## Abstract

**Background:**

Dyspnea is common and severe in intensive care unit (ICU) patients managed for acute respiratory failure. Dyspnea appears to be associated with impaired prognosis and neuropsychological sequels. Pain and dyspnea share many similarities and previous studies have shown the benefit of morphine on dyspnea in patients with end-stage onco-hematological disease and severe heart or respiratory disease. In these populations, morphine administration was safe. Here, we hypothesize that low-dose opioids may help to reduce dyspnea in patients admitted to the ICU for acute respiratory failure. The primary objective of the trial is to determine whether the administration of low-dose titrated opioids, compared to placebo, in patients admitted to the ICU for acute respiratory failure with severe dyspnea decreases the mean 24-h intensity of dyspnea score.

**Methods:**

In this single-center double-blind randomized controlled trial with 2 parallel arms, we plan to include 22 patients (aged 18–75 years) on spontaneous ventilation with either non-invasive ventilation, high flow oxygen therapy or standard oxygen therapy admitted to the ICU for acute respiratory failure with severe dyspnea. They will be assigned after randomization with a 1:1 allocation ratio to receive in experimental arm administration of low-dose titrated morphine hydrochloride for 24 h consisting in an intravenous titration relayed subcutaneously according to a predefined protocol, or a placebo (0.9% NaCl) administered according to the same protocol in the control arm. The primary endpoint is the mean 24-h dyspnea score assessed by a visual analog scale of dyspnea.

**Discussion:**

To our knowledge, this study is the first to evaluate the benefit of opioids on dyspnea in ICU patients admitted for acute respiratory failure.

**Trial registration:**

ClinicalTrials.govNCT04358133. Registered on 24 April 2020.

## Introduction

### Background and rationale {6}

Dyspnea is frequent and intense in intensive care unit (ICU) patients. Dyspnea is present in about half of the patients, whether they are intubated or not [1–5]. In intubated patients, median dyspnea is 50 mm on a visual analog scale ranging from 0 (no dyspnea), to 100 (maximum tolerable dyspnea) [1, 2]. Among non-intubated patients admitted for acute respiratory failure, 55% report moderate to severe dyspnea, and mean dyspnea is 40 mm on a visual analog scale [2, 3, 6].

Such dyspnea has an impact on the comfort, the prognosis, and the quality of life after ICU stay. In ICU patients, dyspnea is strongly and independently associated with anxiety [1–3, 7]. In intubated patients, dyspnea is associated with a longer duration of mechanical ventilation [1]. In patients who receive non-invasive ventilation, the persistence of moderate to severe dyspnea after the first non-invasive ventilation session is associated with higher mortality and longer length of stay [3]. Finally, dyspnea is one of the events participating in the genesis of post-traumatic stress disorder [2].

All these elements plead in favor of a better control of dyspnea in ICU patients. Opioids could find a major place here, as dyspnea and pain are central phenomena [8] with multiple analogies. Both are subjective phenomena and multidimensional sensory experiences with a visceral sensory component and an integrative emotional component. The right insular region appears to be specifically involved in the modulation of dyspneic sensory perception. This same region is activated by nociceptive stimuli [9]. The neurophysiological analogy between dyspnea and pain is thus clearly established by the existence of common afferences [10].

Opioids, whose analgesic properties are well known, also relieve dyspnea [11]. Reciprocally, pain stimulates ventilation, and its control could have beneficial effects on dyspnea [12]. These multiple analogies between dyspnea and pain have motivated the evaluation of the impact of opiates on dyspnea.

In the intensive care unit, the control of dyspnea implies in a first step to correct metabolic abnormalities and in a second step an optimal treatment of the condition causing the acute respiratory failure [7, 13]. In a certain number of patients, dyspnea persists despite all these attempts to relieve it. Opiates could find their place here, modulating the perception of dyspnea by decreasing the intensity of the central ventilatory command and the associated corollary discharge, thus modifying the central perception and possibly decreasing the anxiety [14]. The fear of overdose with respiratory depression has historically been the main obstacle to the widespread use of morphine for the relief of dyspnea in daily clinical practice. However, recent guidelines from the American College of Chest Physicians, the Canadian Thoracic Society [15] and the American Thoracic Society [16] advocate oral or parenteral administration of opioids for refractory dyspnea, which persist despite optimal treatment of the underlying affection.

Several meta-analyses have shown the benefit of morphine on dyspnea, but also its safety, as morphine administration has no impact on blood pressure, PaCO_2,_ or SpO_2_ in patients with end-stage onco-hematological disease, moderate to severe chronic obstructive pulmonary disease, or advanced heart failure [11, 17, 18], and this safety has been confirmed by several expert groups [16]. Randomized and non-randomized studies conducted in patients with terminal cancer, chronic obstructive pulmonary disease, heart failure, or idiopathic fibrosis have shown that morphine was associated with a significant decrease in dyspnea without inducing respiratory depression as suggested by unchanged respiratory rate, tidal volume, blood gas, and end-tidal PaCO_2_ [19–26].

### Objectives {7}

The primary objective of the trial is to determine whether the administration of low-dose titrated opioids, compared to placebo, in patients admitted to the ICU for acute respiratory failure with severe dyspnea decreases the mean 24-h intensity of dyspnea score. The secondary objectives include the evaluation of the impact of the administration of low doses of morphine on the incidence and severity of anxiety and pain every, and the evaluation of signs of intolerance to opioids.

### Trial design {8}

OpiDys is a phase 2 single-center parallel-group double-blind randomized placebo-controlled trial.

## Methods: participants, interventions, and outcomes

### Study setting {9}

**Fig. 1:**
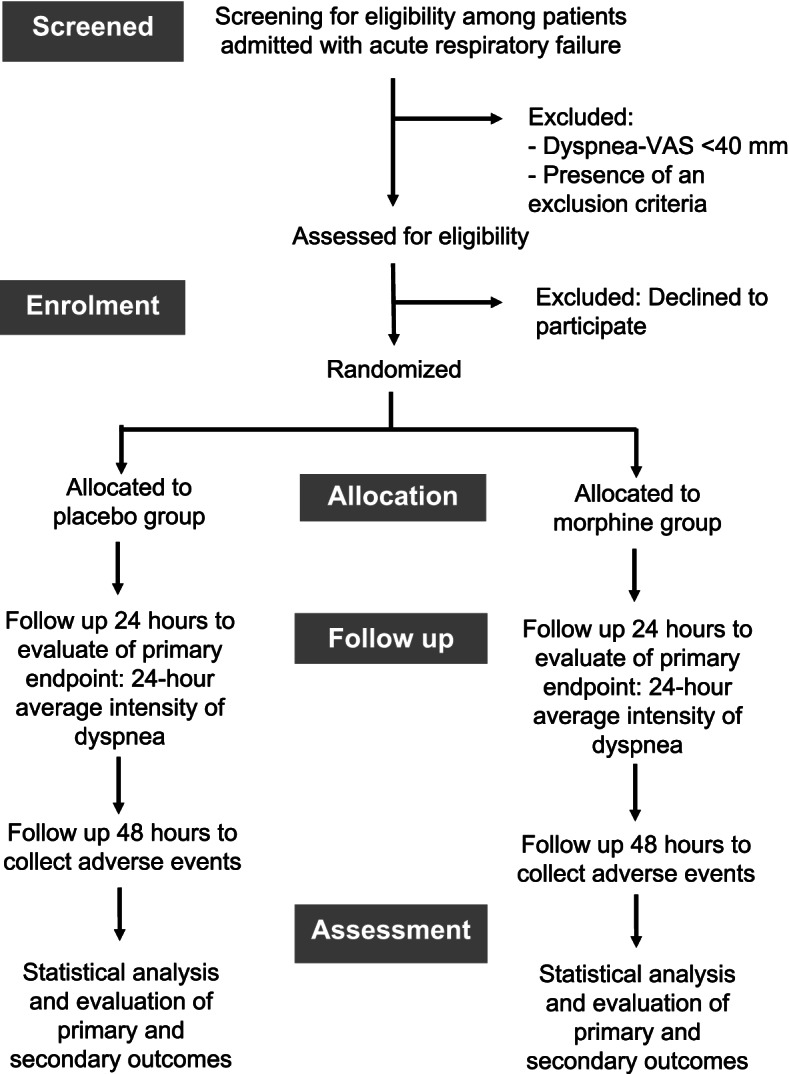
Trial study design

The study will be conducted in a 22 beds medical ICU within the La Pitié-Salpétrière university hospital. See Fig. 1 for the trial study design and Table 1 for the steps of the trial.

**Table 1:** Description of the different steps from the inclusion visit to the end of the study

***Study steps***	***Day 0 (inclusion visit)***	***Day 0 to day 1 (every 4 h)***	***Day 1 (24 h)***	***End of study*** ***Day 2 (48 h)***
***Patient’s information***	***X***			
***Collection of consent***	***X***			
***Medical history***	***X***			
***Randomization***	***X***			
***Clinical examinations and scores***	***X***	***X***	***X***	***X***
***Respiratory support***	***X***	***X***	***X***	***X***
***Tolerance of respiratory support***		***X***	***X***	
***Intravenous titration of morphine/placebo***	***X***			
***Subcutaneous morphine/placebo relay***		***X***		
***Observance***	***X***	***X***	***X***	***X***
***Adverse events***		***X***	***X***	***X***
***Necessity of intubation***		***X***	***X***	***X***
***Nurse’s adhesion/satisfaction about the study***			***X***	

### Eligibility criteria {10}

Inclusion criteria are the followings:

- Adult patients ≤ 75 years,
- Admitted in intensive care for an acute respiratory failure defined as a respiratory rate> 24/min or signs of respiratory distress such as labored breathing or paradoxical inspiration, or SpO2 <90% in ambient air,
- Spontaneous ventilation, either under standard oxygen, high flow oxygen, or non-invasive ventilation,
- Dyspnea ≥40 mm on a Dyspnea-VAS from zero (no dyspnea) to 100 mm (worst possible dyspnea),
- Richmond agitation and sedation scale (RASS) between 0 and 2,
- No confusion, as defined by the Confusion Assessment Method for ICU (CAM-ICU), and
- Signed informed consent of the patient, a relative, or emergency consent process.

Non-inclusion criteria are the followings:

- Intubated patient or intubation planned upon admission,
- Unable to respond to a visual analog scale (hearing or visual impairment, insufficient command of French, previous psychiatric or cognitive disorders known),
- Moribund patient,
- Contraindication to opioids (known hypersensitivity to opioids, severe renal insufficiency (creatinine clearance <30 ml/min), severe hepatocellular insufficiency (factor V <50%)
- Any formal contra-indication of opiates,
- Opioid use within the 24 h before inclusion,
- Pregnancy or breastfeeding,
- Minor and protected adult,
- Exclusion period due to inclusion in another clinical trial,
- Previous inclusion in this study, and
- No affiliation to social security.

### Who will take informed consent {26a}

Prospective patients will be orally informed by the principal investigator and provided with a written information sheet. Signed informed consent will be obtained by the investigator who remains independent from the physician in charge of the patient.

### Additional consent provisions for collection and use of participant data and biological specimens {26b}

Not applicable; no samples were collected.

## Interventions

### Explanation for the choice of comparators {6b}

Morphine hydrochloride is an opioid that is commonly used in hospitals to control pain. It can be administered either intravenously or subcutaneously. The placebo arm will act as a control group for the opioid arm.

### Interventions description {11a}

The experimental group will receive an intravenous titration of morphine at an initial dose of 2 mg, followed by 1 mg every 3 min until Dyspnea-VAS is <40 mm, with a maximum safety dose of 8 mg. Once the target (Dyspnea-VAS <40 mm) or the maximum safety dose is reached, morphine hydrochloride will be relayed subcutaneously (due to the longer half-life of this way of administration). It will initially be administered at a dose of 5 mg immediately at the end of the intravenous titration and then every 4 h for a total of 24 h, according to the dyspnea recorded on the visual analog scales. Dose of subcutaneous morphine will be increased from the previous one by increments of 2.5 mg when the dyspnea evaluated by the visual analog scale is greater than or equal to 40, without exceeding the maximum dose of 10 mg every 4h. It will be reduced in steps of 2.5 mg every 4 h if the dyspnea VAS remains below 40 mm. The total duration of subcutaneous treatment will be 20 h.

The control group will receive a placebo NaCl 0.9% administered according to the same protocol as the experimental arm.

All other treatments will be similar in both groups and will follow national or international guidelines.

### Criteria for discontinuing or modifying allocated interventions {11b}

Morphine hydrochloride will be discontinued in case of nausea ≥ grade 3, constipation ≥ 4, bradypnea < 12/minute, delirium, drowsiness, or alteration of the level of consciousness defined by a Glasgow Coma Scale ≤ 9.

### Strategies to improve adherence to interventions {11c}

**For patients being hospitalized in the ICU, adherence to intervention should be correct.**

### Relevant concomitant care permitted or prohibited during the trial {11d}

All standard practices will be continued throughout the study period. The only intervention prohibited is the administration of opioids other than those given for the need of the protocol.

### Ancillary and post-trial care {30}

Ancillary and post-trial care is not planned.

### Outcomes {12}

#### Primary outcome measure

Average dyspnea over 24 h (Time frame: systematically evaluated every 4 h over 24 h and whenever necessary). Dyspnea will be assessed by VAS-dyspnea (ranging from zero, no dyspnea to 100, worst possible dyspnea), which is a patient-reported outcome (PRO).

#### Secondary outcome measures

- Intensity of dyspnea, every 4 h over 24 h, on a Dyspnea-VAS from 0 to 100 mm (worse) (PRO)
- Incidence of severe dyspnea after the target dyspnea is reached (Dyspnea-VAS ≥40 mm) over 24 h (PRO)
- Intensity of anxiety every 4 h over 24 h, on an Anxiety-VAS from 0 to 100 mm (worse) (PRO)
- Incidence of moderate to severe anxiety (Anxiety-VAS ≥40 mm) over 24 h (PRO)
- Intubation rate within the first 48 h
- Incidence of impaired alertness defined by Glasgow Coma Scale ≤ 12, over 48 h
- Incidence of coma over 48 h
- Incidence of delirium over the first 48 h
- Respiratory rate every 4 h over the first 24 h
- Proportion of patients requiring the transition from one oxygenation technique to another within 24 h
- Intensity of pain every 4 h over 24 h, on a Pain-VAS from 0 to 100 mm (worse)
- Duration of night sleep the first night (PRO)
- Quality of sleep during the first night (PRO) evaluated at the end of the first night with by a VAS from 0 (min) to 100 mm (worse)
- Severity of dry eye (PRO) in the first 24 h evaluated by a VAS from 0 (min) to 100 mm (worse)
- Severity of dry nose (PRO) in the first 24 h evaluated by a VAS from 0 (min) to 100 mm (worse)
- Severity of feeling of gastric distension (PRO) in the first 24 h evaluated by a VAS from 0 (min) to 100 mm (worse)
- Constipation (PRO) in the first 48 h evaluated by a VAS from 0 (min) to 100 mm (worse)
- Nausea (PRO) in the first 48 h evaluated by a VAS from 0 (min) to 100 mm (worse)
- Nurses' adherence to the protocol in the first 24 h evaluated by a VAS from 0 (min) to 100 mm (worse)
- Nurses' satisfaction with the protocol in the first 24 h evaluated by a VAS from 0 (min) to 100 mm (worse)
- Number of non-invasive ventilation sessions in the first 24 h
- Total duration of non-invasive ventilation in the first 24 h
- Tolerance of non-invasive ventilation (PRO) in the first 24 h evaluated by a VAS from 0 (min) to 100 mm (worse)
- Duration of HFNCO (high-flow nasal cannula oxygenation) in the first 24 h
- Tolerance of HFNCO (high-flow nasal cannula oxygenation) in the first 24 h
- Duration of standard oxygen in the first 24 h
- Tolerance of standard oxygen (PRO) in the first 24 h evaluated by a VAS from 0 (min) to 100 mm (worse)
- Any adverse or serious event occurring within the first 48 h, number of adverse events

### Participant timeline {13}

See Table 1 for the participant timeline.

#### Clinical parameters collected at inclusion by the investigator

- Data relating to admission: age, sex, severity score quantified by the Simplified Acute Physiologie Score II (SAPS II), comorbidities, including pre-existing respiratory or cardiac pathology, condition that precipitated the acute respiratory failure responsible for the initiation of ventilatory assistance.
- Physiological values: temperature, blood pressure, respiratory rate, VAS-dyspnea, VAS-anxiety.
- Oxygenation modalities: standard oxygen (flow rate), HFNCO (high-flow nasal cannula oxygenation) (flow rate and inspired fraction of oxygen) or non-invasive ventilation (inspired fraction of oxygen, level of inspiratory assistance, level of positive expiratory pressure); Arterial blood gases: PaO_2_, PaCO_2_, pH, HCO3−, SaO_2_.
- Associated analgesic, anxiolytic or psychotropic treatments administered during the first 24 h.

#### Clinical data collected every 4 h

Dyspnea-VAS (for 24h), Anxiety-VAS (for 24h), Pain-VAS (for 24h), CAM-ICU (for 48h), Glasgow Coma Scale (GCS) (for 48h), respiratory rate (for 24h), change of oxygenation technique (for 48h).

#### Data collected once at 24 h

Intubation, duration of sleep on the first night, quality of sleep on the first night, severity of dry eye, severity of nasal dryness, severity of feeling of gastric distension, constipation, nausea, nurses' adherence to the protocol, nurses’ satisfaction with the protocol, number of NIV sessions, total duration of NIV, NIV tolerance, duration of high-flow oxygen therapy, tolerance of high-flow oxygen therapy, standard oxygen therapy duration, tolerance of standard oxygen therapy, concomitant treatments

#### Clinical data collected once at 48 h

Necessity of intubation, onset of delirium, constipation, nausea.

### Sample size {14}

This is a pilot study. There is very limited data on the intensity of dyspnea in non-intubated patients admitted to the ICU for an acute respiratory failure [3, 6]. Based on these studies, we estimated that mean Dyspnea-VAS over the first 24 h, our primary outcome would be 37 mm in the control arm. We hypothesized that the reduction of mean Dyspnea-VAS over the first 24 h (primary outcome) would be 25 mm, which is 2.5 times the currently admitted minimal clinically important difference (MCID) [27], with a standard deviation of 26 mm. In the experimental arm, mean Dyspnea-VAS over the first 24 h would subsequently be 12 mm. Therefore, with a power of 80%, a one-sided alpha risk of 10% (in order to provide a signal of efficacy without missing a difference), and considering that a non-parametric test will be used, we calculated that 22 patients should be recruited (11 per group).

### Recruitment {15}

The medical ICU of la Pitié-Salpêtrière hospital in which the study is conducted admits annually 1200 patients. Three hundred are admitted for an acute respiratory failure; among them, 150 are not intubated or close to be so on admission. Given the profile of patients admitted in the ICU during the five previous years, we estimated that among these 150 non-intubated patients admitted for acute respiratory failure, 50 will fulfill all the inclusion criteria, among which 35 will fulfill at least one exclusion criteria. Subsequently, we anticipate to include 15 patients per year.

## Assignment of intervention: allocation

### Sequence generation {16a}

The randomization list will be prepared by the clinical trial research unit of the hospital. Randomization ratio will be 1:1. The randomization list will be computer-generated with random permuted blocks. Randomization will be performed after inclusion of the patient on the electronic case report form (eCRF) (Cleanweb, Télémédecine Technologies, Boulogne-Billancourt, France).

### Concealment mechanism {16b}

The administration of the treatment will be done according to the randomization arm following the pre-established randomization list.

Sequentially numbered containers of identical appearance prepared by the pharmacy and containing morphine or placebo will be stored in the ICU. The container with the smallest number available in the department's stock should be assigned to the newly included patient in order to proceed with the randomization.

### Implementation {16c}

After randomization by attribution of the container with the smallest number to the newly included patient, the investigator will report the container number in the eCRF.

## Assignment of interventions: blinding

### Who will be blinded {17a}

Trial participants, researchers involved in the study, and outcome assessors will be blinded. A research nurse who is not involved in the management of patients will be in charge of preparing the treatments from the container. The treatment will be administered by a nurse blinded to the treatment received.

### Procedure for unblinding if needed {17b}

Unblinding will be permissible in case of severe adverse effects.

## Data collection and management

### Plans for assessment and collection of outcomes {18a}

Data will be collected from electronic patient records or by asking the patient and collected with a case report form. Once patients are enrolled, baseline data will be collected. To guarantee consistent assessment, researchers are uniformly trained. The investigator and the study coordinator will be responsible for data collection.

### Plans to promote participant retention and complete follow-up {18b}

No participants will be lost to follow-up as the patients will remain in the ICU until the end of the study.

### Data management {19}

The designated staff will enter the data into the eCRF (Cleanweb, Telemedicine Technologies, Boulogne-Billancourt, France) under the supervision of the principal investigator who is responsible for ensuring that data collected are complete, accurate, and that entry is performed in a timely manner. A data manager will be in charge of checking missing or inconsistent data providing queries to be solved. In case of missing data, the reason will be noted. Corrections, with the reason for the corrections, will also be recorded/tracked in the eCRF.

After resolution of all queries, the database will be locked for statistical analysis.

### Confidentiality {27}

All study-related information will be stored securely at the study site. All participant information will be stored in locked file cabinets in areas with limited access. All reports, data collection, process, and administrative forms will be identified by a coded ID (identification) number only to maintain participant confidentiality. All records that contain names or other personal identifiers, such as locator forms and informed consent forms, will be stored separately from study records identified by code number.

### Plans for collection, laboratory evaluation, and storage of biological specimens for genetic or molecular analysis in this trial/future use {33}

Not applicable, no specimens will be collected.

## Statistical methods

### Statistical methods for primary and secondary outcome {20a}

#### Primary outcome

The comparison between the two treatment groups of the mean dyspnea during the first 24 h will be performed by a Mann Whitney test taking a one-sided alpha risk of 10% to limit the risk of missing a difference. The primary outcome will be analyzed in the modified intent to treat population considering all patients randomized having received at least one dose of intervention in the group in which they will be randomized.

#### Secondary outcomes

Quantitative variables will be compared between the two arms with a Mann-Whitney test. Categorical variables will be compared between the two arms with a chi-square test or a Fisher’s exact test as appropriate.

### Interim analysis {21b}

No interim analysis is planned.

### Methods for additional analyses {20b}

There is no plan to conduct any additional analyses.

### Methods in analysis to handle protocol non-adherence and any statistical methods to handle missing data {20c}

The trial will follow the modified intent to treat principle as defined above. Patients who withdraw consent before having received a dose of treatment will be excluded from statistical analysis. Those patients will be replaced if possible to conduct the statistical analysis on 22 patients, as planned in the sample size calculation. We will not exclude from analysis patients in case of intervention discontinuation and will follow the participants discontinuing the intervention during the study duration. Because of the ICU context, we expect no missing data on the primary outcome.

## Oversight and monitoring

### Composition of the coordinating center and trial steering committee {5d}

This is a single-center study, performed and coordinated in la Pitié-Salpêtrière University Hospital, Paris, France. Day-to-day support for the trial is provided by: the principal investigator (takes supervision of the trial and medical responsibility of the patients), the project manager (trial registration, visits) and the research coordinator (collection of baseline data and follow-up of the patients). The principal investigator and the research coordinator meet weekly.

The research group will act as a trial steering committee. Members of the committee are Pr Alexandre DEMOULE principal investigator, Dr Agnès DECHARTRES methodologist, and Riad BAMEUR and Anne BISSERY URC project managers. The missien of the steering committed are to define the general organization of the research, to coordinate the information, to determine the methodology, to monitor the progress of the research, and to propose methodological adjustments during the course of the research. Members of the steering committee meet before the beginning of the study, three months after the inclusion of the first patient, at the end of recruitment, when the database is frozen, when statistical analyses are statistical analyses, and whenever necessary. The meetings will be face-to-face or by telephone if it is not possible to meet face-to-face.

### Composition of the data monitoring committee, its role, and reporting structure {21a}

Given the fact that this study involves a low-risk intervention, there is no data monitoring committee planned in this study.

### Adverse events reporting and harms {22}

In this study, an adverse event will be defined as any untoward medical occurrence in a patient without regard to the possibility of a causal relationship. The investigators will be responsible for collecting all adverse events in the eCRF based on those referred by the patient spontaneously or by an interview in the follow-up visits. The causality of the adverse event with the intervention will be evaluated and recorded in the medical record and in the eCRF.

The investigators will report to the sponsor all serious adverse events occurring to patients treated in the clinical trial, without undue delay but not later than within 24 h of obtaining knowledge of the events. The sponsor will report to the French Health Products Agency all relevant information about suspected unexpected serious adverse reactions to investigational medicinal products occurring in this clinical trial. Serious adverse events will be defined as events that (1) result in death, (2) are life-threatening, (3) requires prolongation of hospitalization, and (4) result in a significant or lasting disability or handicap.

In addition, the following non-serious adverse events will be of particular interest: nausea ≥grade 3, constipation ≥grade 4, bradypnea <12 per minute, confusion or delirium, drowsiness or even coma, defined by a Glasgow Coma Scale ≤9, pruritus grade ≥4, functional ileus, worsening of the respiratory condition requiring intubation.

### Frequency and plans for auditing trial conduct {23}

The sponsor appoints Clinical Research Assistants (CRA) who ensure that the trial is conducted in accordance with good clinical practice and who carry out regular monitoring visits to research locations. The CRA will check consent forms, compliance with the protocol and the planned procedures, quality of data collected in the eCRF, coherence of data collected with source data, and management of treatments used. The French authority in charge of health products, Agence nationale de sécurité du médicament et des produits de santé (ANSM) can audit the trial at its own discretion.

### Plans for communicating important protocol amendments to relevant parties (e.g. trial participants, ethical committees) {25}

Any modifications to the protocol that impact on the conduct of the study, potential benefit of the patient or may affect patient safety, including changes of study objectives, study design, patient population, sample sizes, study procedures, or significant administrative aspects will require a formal amendment to the protocol. Such amendment will be submitted to the South Mediterranean III Comité de Protection des Personnes Important protocol changes as well as changes in eligibility criteria, outcomes, or analyses will be communicated to the investigators, institutional ethics committee, trial participants, and trial registries.

### Dissemination plans {31a}

The results will be published in a journal of impact and in scientific congresses related to the subject of the study.

## Discussion

We expect this study to have a significant benefit to the patient. Relieving dyspnea should be a major therapeutic goal in ICU patients, as is pain management.

Dyspnea is a nociceptive sensation that shares many characteristics with pain. Patients exposed to dyspnea have reported terrifying experiences. With VAS scores of dyspnea ≥ 40, dyspnea can be described as “moderate to severe.” Similar pain scores would indicate an immediate analgesic response. In addition, patients with dyspnea are more likely to have anxiety than non-dyspnea patients, it is likely that dyspnea may contribute to the severe neuropsychological sequelae of resuscitation.

The expected short-term benefit would be to relieve patients by pharmacological means. The immediate gain for the patient would be the immediate disappearance of this nociceptive sensation, in the same way, that an analgesic treatment relieves pain. In the longer term, the benefit would be to reduce the prevalence and severity of neuropsychological sequelae such as post-traumatic stress disorder. This study will allow us to verify our hypothesis by looking for a signal of effectiveness and to show that a morphine titration protocol is feasible in these patients before the realization of a therapeutic trial of a larger scale.

## Trial status

The current version of the protocol is Version 4.0, of the 22 April.

The first patient included in the study occurred on 16 December 2020.

The recruitment period is 30 months (June 2023).

## Data Availability

The datasets used and/or analyzed during the current study will be available from the corresponding author on reasonable request.
